# Biocontrol of *Penicillium nordicum *Growth and Ochratoxin A Production by Native Yeasts of Dry Cured Ham 

**DOI:** 10.3390/toxins4020068

**Published:** 2012-02-01

**Authors:** Roberta Virgili, Nicoletta Simoncini, Tania Toscani, Marco Camardo Leggieri, Silvia Formenti, Paola Battilani

**Affiliations:** 1 Stazione Sperimentale per l’Industria delle Conserve Alimentari, V.le F. Tanara, 31/A, Parma 43121, Italy; Email: nicoletta.simoncini@ssica.it (N.S.); tania.toscani@ssica.it (T.T.); 2 Institute of Entomology and Plant Pathology, Università Cattolica del Sacro Cuore, Via Emilia Parmense, 84, Piacenza I29100, Italy; Email: marco.camardoleggieri@unicatt.it (M.C.L.); silvia.formenti@unicatt.it (S.F.); paola.battilani@unicatt.it (P.B.)

**Keywords:** antagonistic yeasts, *Penicillium nordicum*, ochratoxin A, NaCl, dry-cured meat product

## Abstract

Twelve yeast strains isolated from the surface of Italian typical dry-cured hams, belonging to *D. hansenii*, *D. maramus*, *C. famata*, *C. zeylanoides* and *H. burtonii* species, and previously selected for their ability to grow in dry-cured ham-like substrates, were screened for antagonistic activity against a toxigenic strain of *P. nordicum* and inhibition of ochratoxin A (OTA) biosynthesis. On average, yeast inhibitory activity was lowered by increasing fungal inoculum and enhanced by NaCl presence. In the assay conditions, *H. burtonii* and *C. zeylanoides* were the most effective, both in inhibiting *P. nordicum* growth and OTA production. *D. hansenii* was the species with the lowest inhibitory activity, especially in the absence of salt. OTA production dropped from the range < LOD − 5000 ppb in *P. nordicum* control plates to the range < LOD − 200 ppb in yeast-added plates. OTA production increased in the presence of NaCl in *P. nordicum* control plates, while salt enhanced inhibition against OTA production in yeast-added plates.

## 1. Introduction

The mycete populations (yeasts and moulds) colonizing the surface layers of dry-cured meat products during maturation have been regarded as positively contributing to the chemosensory properties of final outcome, by means of oxidation prevention and generation of volatile compounds enhancing aroma [1,2]. In the case of dry-cured ham, yeasts form a film on ham surface during early and intermediate processing steps, while moulds can develop fungal mycelium even after yeast growth [3]. 

Among moulds isolated from dry-cured meat facilities and products, Penicillia were largely dominant, and the contamination levels have been found to be associated to the environmental conditions of ripening rooms [4,5], geographic area of production [6,7,8] and seasonality [9]. Among Penicillia, *P. nordicum*, a toxigenic contaminant of protein-rich food [10], was isolated from air and products of dry-curing plants [5,9]. Several *P. nordicum* isolates proved to be important producers of ochratoxin A (OTA), a mycotoxin classified by the International Agency for the Research on Cancer (IARC) as a possible human carcinogen, suspected to be involved in nephropathies and urothelial tract tumours [11]. OTA was detected in substrates contaminated by *P. nordicum* [12], including dry-cured pork samples [13,14]. Currently, the appropriate setting of environmental conditions (temperature, relative humidity and air circulation), is the only accepted tool to prevent the uncontrolled growth of *P. nordicum *inside dry-curing plants. 

Biological control of *P. nordicum* contamination based on microbial antagonists can be investigated as a possible way to improve food safety without affecting sensory quality and properties of typical dry-cured meat products. As a rule, during manufacturing a wild yeast population spontaneously grows on dry-cured ham surface, achieving different counts according to processing stage and type [15,16]. Yeast species isolated from ham during processing mainly belonged to genera *Debaryomyces*, *Candida* and less frequently to *Cryptococcus*, *Rhodotorula *and *Rhodosporidium* [15,16,17]. Native yeasts more frequently isolated from dry-cured hams were regarded as non-pathogenic with the exception of *Candida zeylanoydes* [18] and proved to be able of growing to high populations in conditions of temperature, pH, moisture, a_w_ and salt typical of dry-cured ham surface [1,19]. Recently, selected yeasts were added to dry-cured meat products as starter cultures to enrich the volatile compound profile [2,20]. The antagonistic potential of yeasts against Penicillia has been considered for different food matrixes, such as cereals [21,22], coffee [23], fruits [24] or fermented food like wine [25], cheese and yogurt [26]. Recent studies report the use of antagonistic yeasts as surface starter cultures inhibiting fungal growth also in dry-cured meat products [8,27]. 

The aim of this work is to evaluate *in vitro* the ability of selected yeasts isolated from dry-cured ham surface to compete with *P. nordicum* and to inhibit OTA accumulation in the perspective of using them as surface starter cultures. 

## 2. Materials and Methods

### 2.1. Selection and Identification of Yeast Species

Tested yeast strains (*n* = 12) were selected from a total of 270 isolated from dry-cured ham during the maturing process [15] and stored in the collection of Stazione Sperimentale per l’Industria delle Conserve Alimentari (Parma, Italy). 

The yeast strains, selected in a previous study for their potential competition against *P. nordicum* and ability to grow in a dry-cured ham-like substrate [19], were purified by repeated cultivation on Malt Extract Agar (MEA, Oxoid) and maintained at 4 °C on Yeast Peptone Dextrose agar (YPD yeast extract, 10 g; peptone, 20 g; glucose, 20 g; agar, 15 g and deionized water, 1000 mL) before use. Yeast strains were phenotypically identified as *Candida zeylanoides* (*n* = 2), *Hyphopichia burtonii* (*n* = 2), *Debaryomyces hansenii* (*n* = 2), *Debaryomyces maramus* (*n* = 3) and *Candida famata* (*n* = 3), by means of the Biolog system (Biolog Inc., Hayward, CA, USA). Furthermore, yeast cells from the pure cultures of the above-mentioned strains underwent genetic identification by sequencing the D1/D2 domain of 26rRNA encoding gene [28]. The strains phenotypically identified as belonging to *C. zeylanoides* and *H. burtonii* species were confirmed by genetic system, while the remaining strains were genotyped as *D. hansenii*. 

### 2.2. Preparation of Yeast Inocula

Yeast cultures were grown in 10 mL Malt Extract Broth (MEB, Oxoid) at 25 °C for 48 h. Suspensions were prepared by inoculating 25 mL of YPD broth with a loop full of cells and incubating on a rotary shaker (180 rpm) at 28 °C for 24 h. The density of yeast cultures was determined spectrophotometrically by measuring their optical density at 600 nm. 

### 2.3. Preparation of *P. nordicum* Inocula

An ochratoxin A producing strain of *P. nordicum* isolated from cured pork meat in a previous research [5] and identified at molecular level [29], was used in this study; the strain was stored in the fungal collection of the Institute of Entomology and Plant Pathology, Università Cattolica del Sacro Cuore in Piacenza (code MPVP) and of the Federal Research Centre for Nutrition and Food, in Karlsruhe (Germany; code BFE 838). 

The strain has been grown on Petri dishes (9 cm diameter) with Czapek Yeast extract Agar (CYA: sucrose 30.0 g; yeast extract 5.0 g; sodium nitrate 3.0 g; dipotassium phosphate 1.0 g; magnesium sulfate 0.5 g; potassium chloride 0.5 g; agar 15 g; ferrous sulfate 10 mg and deionized water, 1000 mL) and incubated at 25 °C for 7 days. At the end of incubation, 10 mL of sterilized water was added to the culture surface and it was gently scraped to remove most of the conidia for the inoculum preparation. The obtained conidial suspension was adjusted to 10^2^, 10^4^, 10^6^ conidia/mL using a hemocytometer. 

### 2.4. Inhibition Assay of *P. nordicum* by Yeast Strains

The assay was carried out by means of the test described by Bleve *et al.* [30] modified as follows.

Experiments were performed on YPD agar medium adjusted to pH 6, with and without the addition of sodium chloride (3%). A top agar was prepared by mixing 6 mL of YPD with 0.7% agar and 1 mL of yeast suspension containing 10^8^ cells to obtain a thick, continuous layer on plate surface. This suspension was distributed into Petri plates containing 15 mL of YPD agar media. Then, three 10 μL portions for each *P. nordicum* suspension corresponding to 10^2^, 10^4^, 10^6^ conidia/mL, were separately spotted on each plate and incubated at 20 °C. The inocula of *P. nordicum* were carried out at the same time (co–cultured) and four days after the yeast-media preparation (delayed). Two replicate experiments for each condition were performed. Culture media (YPD and YPD +3% NaCl) plates not inoculated with yeasts were included as control.

Fungal growth was expressed as the average measure (mm) of two orthogonal diameters/colony after 14 days of incubation. The inhibitory activity was calculated by the Equation (1) reported by Lima *et al.* [31]:


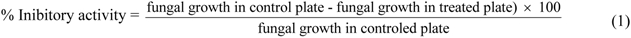


### 2.5. OTA Production by *P. nordicum*

Control and yeast-added plates inoculated with *P. nordicum* were analyzed for OTA content after 14 days of incubation. OTA was measured according to the method of Bragulat *et al.* [32], modified as follows. The content of each plate, including fungal colony and medium, was transferred in a flask with a screw cap, added whit methanol 1:2 (w/v ratio), and left in dark extraction for one hour, shaking each 15 minutes. After filtration, 100 μL of extract was diluted according to Toscani *et al.* [33], analyzing the final solutions by HPLC-FLD quantitative method. It was performed with a C18 column (Waters XTerra^®^, 250 × 2.1 mm, 3 µm) on a Agilent 1100 chromatographic system (Agilent Technologies, USA) under isocratic condition at room temperature, with an aqueous NH_3_/NH_4_Cl (20 mM, pH 9.8):CH_3_CN = 85:15 v/v mobile phase; the flow was 0.2 mL/min and the injected volume was 20 µL. The FLD detection was obtained by means of an Agilent 1100 Fluorescence Detector (Agilent Technologies, USA) (λex = 380 nm, λem = 440 nm). OTA has been expressed as ng/g medium. The limit of detection (LOD) and the limit of quantification (LOQ) in plates were 0.2 ppb and 0.6 ppb respectively.

### 2.6. OTA Recovery in Yeast Treated Plates

Culture media (YPD and YPD +3% NaCl) were spiked with 500 ppb OTA/plate; then, the top agar was added with 1 mL of yeast suspension containing 10^8^ cells in the case of test plates and with 1 mL of physiological solution in the case of control plates. Plates were incubated at 20 °C for 14 days and stored at 20 °C before OTA analysis.

### 2.7. Statistical Analysis

Data analysis was carried out using the SPSS software package (SPSS 15.0, SPSS Inc. Chicago, IL, USA). Data of yeast inhibitory activity and OTA production were inspected for normal distribution using the procedures Frequencies (mean, median, Skewness and Curtosis tests) and Kolmogorov-Smirnov test. Least Square Means (LSM) of yeast inhibitory activity *vs. P. nordicum* were estimated by means of General Linear Model (GLM) procedure for the main effects “yeast species”, “salt presence”, “fungal inoculum level” and their two-way interactions; the Bonferroni *t*-test was used to statistically separate LSM when *P* < 0.05. Percentile distribution of OTA amount in yeast-added- and control plates inoculated with *P. nordicum*, was analyzed using non parametric test (χ2 test), according to yeast species.

## 3. Results

### 3.1. Yeast Inhibition Activity Against *P. nordicum*

The biocontrol test was performed using 12 yeast strains selected among a far exceeding number isolated from dry-cured hams [15]. Yeast selection was made according to their capability of growing in meat model substrates simulating a_w_ values, NaCl content, pH and nutrients of dry-cured ham surface, and exerting *in vitro* competition potential against *P. nordicum* [19]. 

**Figure 1:**
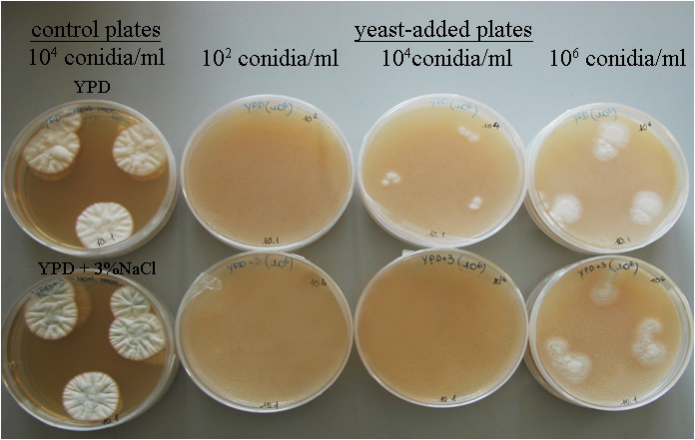
Growth of *P. nordicum* (10^4^ conidia/mL) on Yeast Peptone Dextrose (YPD) and YPD +3% NaCl agar (control) for 7 days at 20 °C compared to the growth of *P. nordicum* (10^2^, 10^4^, 10^6^ conidia/mL) on YPD and YPD +3% NaCl agar containing 10^8^ cfu/mL of selected yeast (yeast-added plates).

For each phenotypic species (*D. hansenii*, *D. maramus*, *C. zeylanoides*, *C. famata*, *H. burtonii*), at least two strains were included in the biocontrol assays, and tested strains caused a visible growth reduction of *P. nordicum* after 14 days of incubation, as displayed in Figure 1. Diameters of fungal colonies were reported in Table 1, both for control- (37–44 mm range) and yeast-added plates (5–11 mm range): differences in *P. nordicum* inoculum were related to colony diameters, because the larger colonies at 14 days of incubation corresponded to higher inocula. 

**Table 1:** Diameters (mm) of *Penicillium nordicum* colonies in control (C) and yeast-added (Y) plates (10^8^ cfu/mL) with Yeast Peptone Dextrose agar after 14 days of incubation at 20 °C. Plates were inoculated with *P. nordicum* at 10^2^, 10^4^ and 10^6^ conidia/mL.

Yeast species	Test	*P. nordicum*^a^		
		10^2^	10^4^	10^6^
*D. hansenii*	C	35.5 ± 2.0	41.6 ± 2.2	42.4 ± 2.4
	Y	12.5 ± 3.5	20.1 ± 3.2	23.7 ± 3.5
*D. maramus*	C	37.1 ± 2.0	41.7 ± 2.2	46.2 ± 1.4
	Y	3.6 ± 1.3	11.4 ± 1.9	18.0 ± 1.8
*C. famata*	C	40.4 ± 1.4	44.4 ±1.4	46.6 ± 1.4
	Y	7.1 ± 1.4	14.0 ± 2.8	15.9 ± 2.1
*C. zeylanoides*	C	34.4 ± 1.9	39.6 ± 2.0	41.7 ± 2.1
	Y	2.1 ± 1.0	5.7 ± 2.2	8.8 ± 2.3
*H. burtonii*	C	35.4 ± 2.2	39.5 ± 2.0	41.9 ± 2.1
	Y	0.7 ± 0.4	5.6 ± 1.8	8.8 ± 1.8

^a^ Colony diameter is reported as mean ± standard error.

Inhibitory activity against *P. nordicum* was not influenced by yeast co- or delayed inoculation (data not shown). A different inhibitory activity against *P. nordicum* was found among yeast species (Table 2): *C. zeylanoides* and *H. burtonii* showed the highest inhibition (86% and 88% respectively) and *D. hansenii* the lowest one (58%).

**Table 2:** Effect of yeast species, salt presence in YPD medium and fungal inoculum concentrations over yeast inhibitory activity *vs. P. nordicum *after 14 days of incubation at 20 °C. Least Square Means of % inhibitory activity according to yeast species (YS), *P. nordicum *inoculum concentration (PN), and NaCl presence in YPD (S) were reported ^a^.

Item	% Inhibitory activity		*P*-value
**YS**			*0.000*
*D. hansenii*	58.2 c		
*D. maramus*	75.9 b		
*C. famata*	72.6 b		
*C. zeylanoides*	85.9 a		
*H. burtonii*	87.9 a		
**PN**			*0.000*
10^2^ conidia/mL	87.9 a		
10^4^ conidia/mL	73.2 b		
10^6^ conidia/mL	67.1 b		
**S**			*0.000*
0% NaCl	71.7 b		
3% NaCl	80.5 a		
Interaction YS × S			*0.000*
Interaction YS × PN			0.724
Interaction S × PN			0.803

^a^ different letters in the same column mean significant differences (Bonferroni multiple comparison test, *P* < 0.05).

Assayed yeast inhibitory activity was affected by the concentration of *P. nordicum* inoculum: the growth of colonies from the lowest inoculum concentration (10^2^ conidia/mL) was the most inhibited by antagonistic yeasts; similar growth was observed in colonies grown 10^4^ and 10^6^ conidia/mL as inoculum.

Salt is the basic ingredient of dry-cured meat products and its influence over yeast biocontrol was tested by means of NaCl addition in YPD medium. The presence of 3% NaCl increased (*P* < 0.05) the inhibitory activity of yeasts against *P. nordicum*, and a significant interaction between salt and yeast species has been found (Table 2).

**Figure 2:**
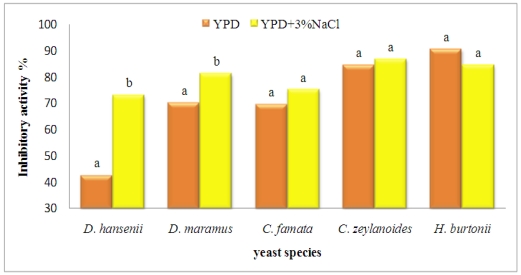
Influence of NaCl (0% or 3% in YPD medium) on yeast inhibitory activity against *P. nordicum* (% diameter reduction at 14 days of incubation at 20 °C).

As a consequence, the influence of salt over yeast inhibitory activity changed according to yeast species, as displayed in Figure 2. Salt enhanced significantly the inhibition exerted by the halophilic yeast *D. hansenii* against *P. nordicum* (70% *vs.* 40% in YPD with and without salt respectively). 

### 3.2. OTA Production in Control and Yeast-Added Plates

Ochratoxin A production by *P. nordicum* was quantified both in yeast-added and control plates after 14 days of incubation (Table 3): OTA values were grouped according to *P. nordicum* inoculum concentrations, and spanned in the range < LOD – 5000 ppb. In control plates, OTA values associated to the lowest *P. nordicum* inoculum exceeded those found with higher inoculum concentrations. In plates with antagonist yeasts, featured by a sharp OTA reduction, the effect of fungal inoculum level was not so clear. 

**Table 3:** Mean, median and 95^th^ percentile production of ochratoxin A (OTA) (ppb) by *P. nordicum* in control and yeast-added plates after 14 days of incubation. OTA values were averaged over tested yeast strains.

		OTA (ppb)		
		Mean	Median	95^th^ percentile
Control plates three levels of *P. nordicum *inoculum as conidia/mL	10^2^	2353	2518	4523
	10^4^	1652	1587	3429
	10^6^	1064	1000	2432
Yeast-added plates three levels of *P. nordicum *inoculum as conidia/mL + 10^8^ cfu/mL yeasts (Y)	10^2^ + Y	33.3	4.0	187
	10^4^ + Y	35.2	4.0	138
	10^6^ + Y	42.7	7.5	179

According to OTA value distribution, three percentile groups of equal frequency (33.3%) were created and named high (H), intermediate (M) and low (L) with reference to OTA content range (Table 4). 

**Table 4:** Distribution (%) of OTA amounts detected in control (C) and yeast-added (Y) plates into groups based on OTA percentile values with 3 cut points for equal groups (33.3%) ^a^.

*Yeast species*	OTA	C	Y
*D. maramus*	L	0	69
	M	29	31
	H	71	0
*D. hansenii*	L	0	47
	M	35	53
	H	65	0
*C. famata*	L	0	58
	M	40	42
	H	60	0
*C. zeylanoides*	L	0	75
	M	35	25
	H	65	0
*H. burtonii*	L	0	86
	M	33	14
	H	67	0

^a^ Low (L) range = < LOD – 27 ppb; Intermediate (M) range = 28–911 ppb; High (H) range = 912–4697 ppb.

A comparison was carried out between control and yeast-added plates to compare percentage distributions between groups (χ2 statistic). The yeast inhibitory activity over OTA production was significant (*P* < 0.001): control plates were mostly allocated to H group, the yeast-added ones to L group, while the frequencies of control and treated plates in M group were similar.

The effect of yeast species over OTA distribution in H, M and L groups is reported in Table 5: (i) the frequency distribution of *H. burtonii* differed from *D. maramus*, *C. famata* and *D. hansenii*; (ii) *C. zeylanoides* differed from *C. famata* and *D. hansenii*; (iii) *D. maramus* differed from *D. hansenii*. 

**Table 5:** P values (χ2 statistic, *P* < 0.05 means significant difference) for the effect of yeast species on OTA distribution into groups reported in Table 4.

	*D. maramus *	*C. famata *	*C. zeylanoides *	*H. burtonii *
*D. hansenii *	*0.000*	0.310	*0.000*	*0.000*
*D. maramus *		0.092	0.260	*0.032*
*C. famata *			*0.006*	*0.000*
*C. zeylanoides *				0.135

**Figure 3:**
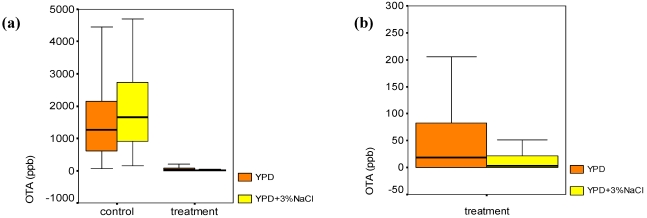
(**a**) Ochratoxin A (OTA) distribution in control and yeast treated plates, clustered according to salt presence in growing media (YPD and YPD +3% NaCl); (**b**) Magnification of yeast treated plate box plots.

Ochratoxin A values detected in control and yeast-treated plates were split with reference to salt presence in YPD medium (Figure 3). In control plates, salt presence favored OTA production (Figure 3a), while in yeast-added plates, an opposite result was evidenced (Figure 3b). 

OTA analysis in spiked control and yeast-added plates had the same % recovery, showing that yeast presence did not affect OTA extraction from the growing medium (Table 6). 

**Table 6:** Detected amount and % recovery of ochratoxin A (OTA) in plates without (N) or with 10^8^ cfu/mL yeasts (Y). Each plate was spiked with 500 ppb OTA. OTA values are reported as mean ± standard deviation.

medium	Yeasts (cfu/mL)	OTA (ppb)	Recovery (%)
YPD	N	405 ± 25	81
	Y	375 ± 17	75
YPD +3% NaCl	N	390 ± 15	78
	Y	425 ± 13	85

## 4. Discussion

The microbial community growing on dehydrated surface of dry-cured ham during the maturing phase is dominated by several species of yeasts and moulds [15,34]; the former population, is present from early processing steps, while the latter becomes dominating during maturation [4]. The efficacy of native yeasts as biocontrol agents is questionable because moulds frequently colonize ham surface in the presence of yeasts [3].

Information on the antagonistic activities of yeasts isolated from dry-cured meat products against toxigenic fungi is scarce [35]: recent study differentiated yeasts isolated from dry-cured ham according to their inhibitory effect *in vitro* against an OTA-producing *P. nordicum* strain [19]. Although the mechanisms underlying the observed inhibition were not yet investigated, it is likely that the presence of a high yeast population (10^8^ cfu/mL) may restrict the availability of nutrients and sites for colonization, essential for the germination of mould spores [36]. Significant differences were found among assayed yeasts in terms of % inhibitory activity, being *H. burtonii* and *C. zeylanoides* the most, and *D. hansenii* the least effective in inhibiting *P. nordicum* growth. 

*D. hansenii* is the predominant species in dry-cured ham isolates during processing [15,17], but this predominance becomes more remarkable in fully matured products. The high occurrence of *D. hansenii* in dry-cured meat products is most probably due to its moderately halophilic properties, accounting for its optimal growth at 3–5% salt [37]. A recent study demonstrated that a higher number of *D. hansenii* strains isolated from dry-cured meat products were able to growth up to 15% NaCl if compared to *C. zeylanoides* [16]. However, the lowest inhibitory activity exerted in the present study by *D. hansenii* against *P. nordicum* could be predictive of less effectiveness of this yeast as biocontrol agent. 

The occurrence of *C. zeylanoides* was found to decrease from early to late processing steps [16,17]: in Italian dry-cured hams, though isolated up to final maturation, the incidence on muscle surface was lower during maturing phases than at the beginning of processing [15]. So, even if *in vitro* tested strains of *C. zeylanoides* highly inhibited *P. nordicum*, under dry-curing plant conditions (fluctuation of temperature, relative humidity, air circulation, decrease of water availability in surface muscle), *C. zeylanoides*, having been found to fail in maintaining high populations in matured dry-cured ham, could have less ecological fitness than *D. hansenii*. Furthermore, *C. zeylanoides* was included among emerging pathogenic yeasts [18] and considered unsuitable as biocontrol agent.

*Hyphopichia burtonii*, referred to as “yeast-like fungi”, was isolated from dry-cured hams after surface fat application during maturation. The yeast was probably introduced by the rice flour mixed with the spreadable pork fat (sugna) used at mid maturing to prevent excessive dehydration of ham surface [15]. It was reported as capable of inhibiting the spore germination of *P. verrucosum* and the growth of *P. roquefortii* [22,38], and the interest in this species is confirmed by the performance against *P. nordicum* showed in the present study. Otherwise, the attribution of *H. burtonii* to dry-cured ham native yeasts is questionable and its potential as biocontrol agent against *P. nordicum* should be tested in ripening plant conditions, before sugna application. 

*Debaryomyces maramus* and *C. famata* (the anamorphic form of *D. hansenii*) were isolated throughout the processing of Parma ham and other traditional Greek and Spanish dry-cured products [15,39,40], even less frequently than *D. hansenii* and *C. zeylanoides*. In the case of orange fruits, a *C. famata* population (10^8^ cfu/mL) gave a successful biocontrol toward *Penicillium digitatum* [41] in the present study *C. famata* exerted lower inhibition against *P. nordicum* than *H. burtonii* and *C. zeylanoides*, and an higher inhibition than *D. hansenii*. *Penicillium* contamination in cured meat production facilities and products was found to be affected by a large variability [4,42]: accordingly, the biocontrol test was performed using inoculation levels of *P. nordicum* corresponding to 10^2^, 10^4^, 10^6^ conidia/mL. Yeast inhibitory activity was affected by fungal concentration: at the lowest inoculum level, yeast biocontrol was more effective (*P* < 0.05), showing that the concentration of the mould is a key factor for yeast effectiveness. As a consequence, in future *in vivo *applications of antagonistic yeasts for dry-cured ham biocontrol, it will be important to keep the population of contaminating moulds as low as possible, in agreement with processing procedures for high quality dry-cured meat products aimed at this specific target [43]. Among yeast dominant species isolated from dry-cured hams, the tolerance to NaCl has been evidenced [16,19], a property enabling autochthonous yeasts to colonize and prevail in dry-cured ham surface microflora: this halotolerance is in agreement with the positive effect of 3% NaCl presence upon yeast inhibition activities. The same tolerance was recently demonstrated also for *P. nordicum*, capable of growing and producing OTA in dry-cured pork samples in the presence of 5% NaCl and 0.90 a_w_ [14]. A significant interaction “yeast × salt” was found and displayed in Figure 2. The inhibitory activity was significantly enhanced by 3% NaCl only for *Debaryomyces* spp., mostly in the case of *D. hansenii*. 

The tested strains of *D. hansenii* gave, on average, the least inhibition among tested yeasts, but, thanks to its halophilic aptitude [37], may be that NaCl had a key role in inhibition expression. Some authors found that *D. hansenii* was able to synthesize toxic proteins with killer capacity against sensitive strains, but the lethal activity of these toxins was demonstrated or increased only in the presence of NaCl [44,45]. Other tested yeasts did not show any difference due to NaCl presence in the medium. 

The biocontrol by means of antagonistic yeasts is a potential strategy both against toxigenic moulds and to prevent or minimize mycotoxin production. In this study, the production of OTA was examined when *P. nordicum* was in pure culture (control plates) or cultured with yeast strains (yeast-added plates). The temperature of the biocontrol test (20 °C) was previously found suitable for OTA high production in dry-cured pork model systems [14]. In the presence of yeasts, the growth inhibition of *P. nordicum* correlated with the sharp reduction of OTA (Table 3). In the above-mentioned test conditions, the competition for nutrients and space could be the mechanism inhibiting both *P. nordicum* growth and secondary metabolism, including OTA production [30]. In yeast-added plates, an average tendency of OTA production to increase according to *P. nordicum* inoculum concentration was found. In control plates, when the *P. nordicum* inoculum is low (10^2^ conidia/mL), the nutrients not required for fungal growth could be used for OTA biosynthesis. Ochratoxin A frequency distribution into groups of equal frequency (33.3%) L, M and H changed according to yeast presence and species. Treated plates corresponded to low (L) or intermediate (M) OTA category production, while control plates fell mostly in high (H) category. In agreement with inhibitory activity values reported in Table 2, *D. hansenii* gave OTA value frequency different from *H. burtonii*, *C. zeylanoides* and *D. maramus*, *C. famata* differed from *H. burtonii* and *C. zeylanoides* and *D. maramus* from *H. burtonii*. These results suggested that the same mechanism of biocontrol was effective against both *P. nordicum* growth and OTA production; otherwise, other studies reported that although the growth of spoilage fungi is inhibited by biocontrol of antagonistic yeasts, their metabolic activities are not necessarily reduced [21,36]. The effect of 3% NaCl in control and yeast-added plates was examined with reference to OTA production (Figure 3): in control plates with NaCl, OTA reached higher values than in without-salt counterparts, suggesting a salt-stress related increase in mycotoxin biosynthesis. Otherwise, the presence of NaCl improved yeast biocontrol, enabling an almost total inhibition of OTA production. This finding is in agreement with the “NaCl” positive effect against *P. nordicum* growth in yeast-added plates (Table 2). Yeasts were also reported for decreasing OTA content by adsorbption on the external and internal part of the cells, sequestering the toxin or even degrading it [46]; the cell wall has a chemical composition that varies from yeast to yeast, regulating the adsorption/non-adsorption activity of yeasts [47]. The assay made to check OTA stability and recovery in control and yeast-added plates (Table 6), shows that OTA was stable in YPD medium with and without NaCl, and OTA recovery was not affected by yeast presence. In this respect, the sharp decrease of OTA in treated plates cannot be ascribed to degradation or adsorption by yeasts. Moreover, even in the case of adsorption, the usage of methanol for extraction, by denaturing yeast parietal protein, allowed OTA to be recovered (75–85% recovery range).

## 5. Conclusions

This study demonstrated that selected strains of yeasts isolated from dry-cured ham, exerted *in vitro* inhibition activity against *P. nordicum* cultured in the presence of high yeast population (10^8^ cfu/mL). The inhibition was effective against both fungal growth and OTA production, and was significantly influenced by yeast species, level of fungal contamination and NaCl presence in culture medium. 

NaCl promoted a significant increase of biocontrol activity, favoring the most halophilic yeast strains such as *D. hansenii*, but other species exerted a more efficient control on *P. nordicum* growth and OTA synthesis. The antagonistic activity of tested yeasts was negatively affected by increasing levels of fungal contamination. Assayed yeasts could be effective biocontrol agents for dry-cured meat products, if capable of quickly growing and long lasting. In this respect, future works will be aimed to test the efficacy of different yeasts species in ripening plants, to optimize the inoculum distribution and to achieve suitable conditions for yeast stable and efficient colonization of product surface.

## References

[1] Martin M., Cordoba J.J., Aranda E., Cordoba M.G., Asensio M.A. (2006). Contribution of selected fungal population to the volatile compounds of dry -cured ham.. Int. J. Food Microbiol..

[2] Andrade M.J., Cordoba J.J., Sánchez B., Casado E.M., Rodríguez M. (2009). Evaluation and selection of yeasts isolated from dry-cured Iberian ham by their volatile compound production.. Food Chem..

[3] Spotti E., Berni E., Cacchioli C., Toldrá F. (2008). Characteristics and Applications of Molds.. Meat Biotechnology.

[4] Asefa D.T., Kure C.F., Gjerde R.O., Omer M.K., Langsrud S., Nesbakken T., Skaar I. (2010). Fungal growth pattern, sources and factors of mould contamination in dry-cured meat production process. Int. J. Food Microbiol..

[5] Battilani P., Pietri A., Giorni P., Formenti S., Bertuzzi T., Toscani T., Virgili R., Kozakiewicz Z. (2007). *Penicillium *population in dry-cured ham manufacturing plants.. J. Food Prot..

[6] Nunez F., Rodriguez M.M., Bermudez M.E., Cordoba J.J., Asensio M.A. (1996). Composition and toxigenic potential of the mould population on dry-cured Iberian ham.. Int. J. Food Microbiol..

[7] Comi G., Orlic S., Redzepovic S., Urso R., Iacumin L. (2004). Moulds isolated from Istrian dried ham at the pre-ripening and ripening level.. Int. J. Food Microbiol..

[8] Wang X., Ma P., Jiang D., Peng Q., Yang H. (2006). The natural microflora of Xuanwei ham and the no-mouldy ham production.. J. Food Eng..

[9] Castellari C., Quadrelli A.M., Laich F. (2010). Surface mycobiota on Argentinean dry fermented sausages.. Int. J. Food Microbiol..

[10] Larsen T.O., Svendsen A., Smedsgaard J. (2001). Biochemical characterization of ochratoxin A-producing strains of the genus *Penicillium*.. Appl. Environ. Microbiol..

[11] Pfohl-Leszkowicz A. (2009). Ochratoxin A and aristolochic acid involvement in nephropathies and associated urothelial tract tumors.. Arh. Hig. Rada Toksikol..

[12] Frisvad J.C., Samson R.A. (2004). Polyphasic taxonomy of *Penicillium *subgenus *Penicillium*: A guide to identification of food and air-borne terverticillate Penicillia and their mycotoxins.. Stud. Mycol..

[13] Pietri A., Bertuzzi T., Gualla A., Piva G. (2006). Occurrence of ochratoxin A in raw ham muscles and in pork products from northern Italy.. Ital. J. Food Sci..

[14] Battilani P., Formenti S., Toscani T., Virgili R. (2010). Influence of abiotic parameters on ochratoxin A production by *Penicillium nordicum *strain in dry-cured meat model systems.. Food Control.

[15] Simoncini N., Rotelli D., Virgili R., Quintavalla S. (2007). Dynamics and characterization of yeasts during ripening of typical Italian dry-cured ham.. Food Microbiol..

[16] Asefa D.T., Møretrø T., Gjerde R.O., Langsrud S., Kure C.F., Sidhu M.S., Nesbakken T., Skaar I. (2009). Yeast diversity and dynamics in the production processes of Norwegian dry-cured meat products.. Int. J. Food Microbiol..

[17] Nunez F., Rodriguez M.M., Cordoba J.J., Bermudez M.E., Asensio M.A. (1996). Yeast population during ripening of dry-cured Iberian ham.. Int. J. Food Microbiol..

[18] Levenson D., Pfaller M.A., Smith M.A., Hollis R., Gerarden T., Tucci C.B., Isenberg H.D. (1991). *Candida zeylanoides*: an opportunistic yeast.. J. Clin. Microbiol..

[19] Simoncini N., Virgili R., Quintavalla S., Formenti S., Battilani P. (2009). Biotypization of autochthonous yeasts of dry-cured meat products.. Ind. Conserve.

[20] Pinna A., Quintavalla S., Simoncini N., Toscani T., Virgili R. (2009). Volatile organic compounds of a ham-like model system inoculated with authoctonous yeasts isolated from typical hams.. Ind. Conserve.

[21] Petersson S., Hansen M.W., Axberg K., Hult K., Schnürer J. (1998). Ochratoxin A accumulation in cultures of *Penicillium verrucosum* with the antagonistic yeast *Pichia anomala* and *Saccharomyces cerevisiae*.. Mycol. Res..

[22] Druvefors U.A., Schnürer J. (2005). Mold-inhibitory activity of different yeast species during airtight storage of wheat grain.. FEMS Yeast Res..

[23] Masoud W., Kaltoft C.H. (2006). The effects of yeasts involved in the fermentation of Coffea arabica in East Africa on growth and ochratoxin A (OTA) production by *Aspergillus ochraceus*.. Int. J. Food Microbiol..

[24] Guinebretiere M.H., Nguyen-The C., Morrison N., Reich M., Nicot P. (2000). Isolation and characterization of antagonists for the biocontrol of the postharvest wound pathogen *Botrytis cinerea* on strawberry fruits.. J. Food Prot..

[25] Suzzi G., Romano P., Ponti I., Montuschi C. (1995). Natural wine yeasts as biocontrol agents.. J. Appl. Bacteriol..

[26] Liu S.Q., Tsao M. (2009). Biocontrol of dairy moulds by antagonistic dairy yeast *Debaryomyces hansenii* in yoghurt and cheese at elevated temperatures.. Food Control.

[27] Sánchez-Molinero F., Arnau J. (2008). Effect of the inoculation of a starter culture and vacuum packaging (during resting stage) on the appearance and some microbiological and physicochemical parameters of dry-cured ham.. Meat Sci..

[28] Kurtzman C.P., Robnett C.J. (1997). Identification and phylogeny of ascomycetous yeasts from analysis of nuclear large-subunit (26S) ribosomal DNA partial sequences.. Antoine van Leeuwenhoek.

[29] Bogs C., Battilani P., Geisen R. (2006). Development of a molecular detection and differentiation system for ochratoxin A producing *Penicillium *species and its application to analyse the occurrence of *P. nordicum* in cured meats.. Int. J. Food Microbiol..

[30] Bleve G., Grieco F., Logrieco A., Visconti A. (2006). Isolation of epiphytic yeasts with potential for biocontrol of *Aspergillus carbonarius* and *A. niger* on grape.. Int. J. Food Microbiol..

[31] Lima G., Arru S., De Curtis F., Arras G. (1999). Influence of antagonistic, host fruit and pathogen on the biological control of postharvest fungal diseases by yeasts. J. Ind. Microbiol. Biotechnol..

[32] Bragulat M.R., Abarca M.L., Cabañes F.J. (2001). An easy screening method for fungi producing ochratoxin A in pure culture.. Int. J. Food Microbiol..

[33] Toscani T., Moseriti A., Dossena A., Dall’Asta C., Simoncini N., Virgili R. (2007). Determination of ochratoxin A in dry-cured meat products by HPLC-FLD quantitative methods.. J. Chromatogr. B.

[34] Asefa D.T., Gjerde R.O., Sidhu M.S., Langsrud S., Kure C.F., Nesbakken T., Skaar I. (2009). Moulds contaminants on Norwegian dry-cured meat products.. Int. J. Food Microbiol..

[35] Spotti E., Berni E., Cacchioli C., Simoncini N., Quintavalla S. (2009). Growth and antagonistic activity of *Hyphopichia burtonii* against other fungal species frequently found on meat products during maturation.. Ind. Conserve.

[36] Björnberg A., Schnürer J. (1993). Inhibition of the growth of the grain-storage molds in vitro by the yeast *Pichia anomala *(Hansen) Kurtzman.. Can. J. Microbiol..

[37] Breuer U., Harms H. (2006). *Debaryomyces hansenii*—an extremophilic yeast with biotechnological potential.. Yeast.

[38] Ramakrishna N., Lacey J., Smith J.E. (1996). Colonization of barley grain by *Penicillium verrucosumand* Ochratoxin A formation in the presence of competing fungi.. J. Food Prot..

[39] Metaxopoulos J., Stavropoulos S., Kakouri A., Samelis J. (1996). Yeasts isolated from traditional Greek dry salami.. Ital. J. Food Sci..

[40] Monte E., Villanueva J.R., Domìnguez A. (1986). Fungal profiles of Spanish country-cured hams.. Int. J. Food Microbiol..

[41] Arras G. (1996). Mode of action of an isolate of* Candida famata *in biological control of *Penicillium digitatum* in orange fruits.. Postharvest Biol. Technol..

[42] Sonjak S., Ličen M., Frisvad J.C., Gunde-Cimerman N. (2011). The mycobiota of three dry-cured meat products from Slovenia.. Food Microbiol..

[43] Asefa D., Kure C.F., Gjerde R.O., Langsrud S., Omer M.O., Nesbakken T., Skaar I. (2010). A HACCP plan for mycotoxigenic hazards associated with dry-cured meat production processes.. Food Control.

[44] Llorente P., Marquina D., Santos A., Peinado J.M., Spencer-Martins I. (1997). Effect of salt on the killer phenotype of yeasts from olive brines.. Appl. Environ. Microbiol..

[45] Marquinia D., Barroso J., Santos A., Peinado J.M. (2001). Production and characteristics of *Debaryomyces hansenii* killer toxin.. Microbiol. Res..

[46] Patharajan S., Reddy K.R.N., Spadaro D., Lore A., Gullino M.L., Garibaldi A., Karthikeyan V. (2011). Potential of yeast antagonists on *in vitro* biodegradation of ochratoxin A.. Food Control.

[47] Caridi A. (2007). New perspectives in safety and quality enhancement of wine through selection of yeasts based on the parietal adsorption activity.. Int. J. Food Microbiol..

